# Supplementary material for: GEF-H1 Transduces FcεRI Signaling in Mast Cells to Activate RhoA and Focal Adhesion Formation during Exocytosis
Source: Cells. 2023 Feb 7;12(4):537. doi: 10.3390/cells12040537 (PMC9954420; doi:10.3390/cells12040537)
Supplement: Supplementary file 1 [file cells-12-00537-s001.zip › cells-2068935-supplementary.pdf]

Guo et al., 2023 Supplementary Figure S1

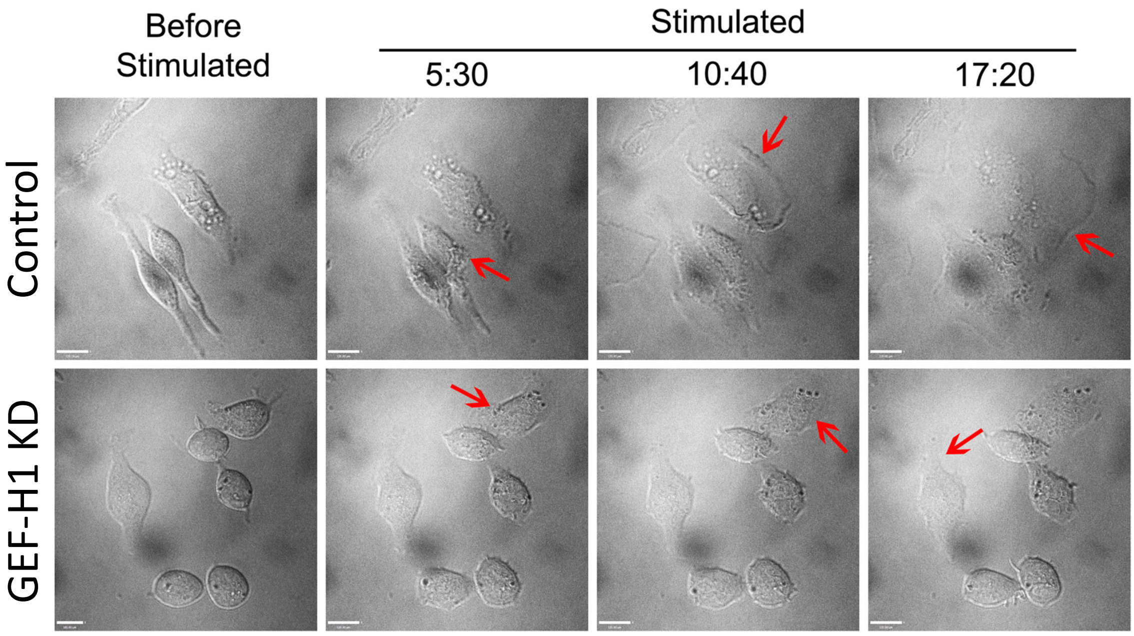

Figure S1. Depletion of GEF-H1 does not block membrane ruffling and lamellipodia formation. Membrane ruffling and lamellipodia formation was visualized in live cells during antigen stimulation using Differential Interference Contrast (DIC) microscopy. Shown are representative still images of time points before and after antigen stimulation from **Video S3** of control cells and **Video S4** of GEF-H1 depleted cells (GEF-H1 KD). The formation of membrane ruffling and lamellipodia formation occurred in control and GEF-H1 depleted cells (*red arrows*). Cell spreading was reduced in GEF-H1 depleted cells. Scale bar = 10 µm.

Supplementary videos showing live-cell DIC imaging are available at the following direct object identifiers:

Video S3: Differential Interference Contrast (DIC) live-cell imaging of antigen-stimulated RBL-2H3 cells, control

https://doi.org/10.6084/m9.figshare.21454953

Video S4: Differential Interference Contrast (DIC) live-cell imaging of antigen-stimulated RBL-2H3 cells, GEF-H1 knockdown

https://doi.org/10.6084/m9.figshare.21455010

Guo et al., 2023 Supplementary Figure S2

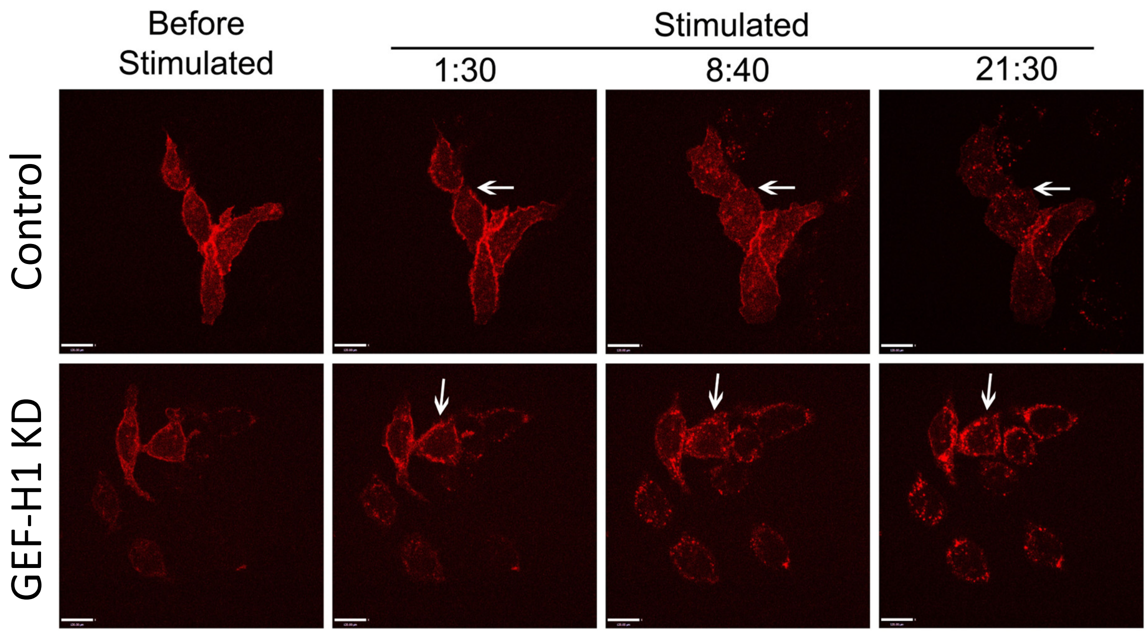

Figure S2. Depletion of GEF-H1 does not affect F-actin remodeling at the cell periphery. Actin remodeling was visualized in live cells during antigen stimulation using cells transfected with the F-actin probe Lifeact-mRuby. Shown are representative still images of time points before and after antigen stimulation from **Video S5** of control cells and **Video S6** of GEF-H1-depleted cells (GEF-H1 KD). When antigen-stimulated, control and GEF-H1 depleted cells formed F-actin rich lamellipodia at the leading edge (*white arrow*). Scale bar = 10 µm.

Supplementary videos showing live-cell imaging of Lifeact-mRuby are available at the following direct object identifiers:

Video S5: Lifeact-mRuby live-cell imaging of antigen-stimulated RBL-2H3 cells, control

https://doi.org/10.6084/m9.figshare.21455076

Video S6: Lifeact-mRuby live-cell imaging of antigen-stimulated RBL-2H3 cells, GEF-H1 knockdown

https://doi.org/10.6084/m9.figshare.21455112
